# Stationary stable cross-correlation pattern and task specific deviations in unresponsive wakefulness syndrome as well as clinically healthy subjects

**DOI:** 10.1371/journal.pone.0300075

**Published:** 2024-03-15

**Authors:** David E. Apablaza-Yevenes, María Corsi-Cabrera, Antonieta Martinez-Guerrero, Georg Northoff, Caterina Romaniello, Marina Farinelli, Erik Bertoletti, Markus F. Müller, Zeidy Muñoz-Torres

**Affiliations:** 1 Instituto de Ciencias Básicas y Aplicadas, Universidad Autónoma del Estado de Morelos, Morelos, México; 2 Unidad de Investigación en Neurodesarrollo, Instituto de Neurobiología, Universidad Nacional Autónoma de México, Querétaro, México; 3 Institute of Mental Health Research, University of Ottawa, Ottawa, Ontario, Canada; 4 Center for Cognition and Brain Disorders, Hangzhou Normal University, Hangzhou, People’s Republic of China; 5 Mental Health Centre, Zhejiang University School of Medicine, Hangzhou, People’s Republic of China; 6 Santa Viola Hospital, Colibrì Consortium, Bologna, Italy; 7 Villa Bellombra Hospital, Colibrì Consortium, Bologna, Italy; 8 Centro de Ciencias de la Complejidad (C3), Universidad Nacional Autónoma de México, Ciudad de México, México; 9 Centro de Investigación en Ciencias, Universidad Autónoma del Estado de Morelos, Morelos, México; 10 Centro Internacional de Ciencias A.C., Morelos, México; 11 Facultad de Psicología, Universidad Nacional Autónoma de México, Ciudad de México, México; Waseda University, JAPAN

## Abstract

Brain dynamics is highly non-stationary, permanently subject to ever-changing external conditions and continuously monitoring and adjusting internal control mechanisms. Finding stationary structures in this system, as has been done recently, is therefore of great importance for understanding fundamental dynamic trade relationships. Here we analyse electroencephalographic recordings (EEG) of 13 subjects with unresponsive wakefulness syndrome (UWS) during rest and while being influenced by different acoustic stimuli. We compare the results with a control group under the same experimental conditions and with clinically healthy subjects during overnight sleep. The main objective of this study is to investigate whether a stationary correlation pattern is also present in the UWS group, and if so, to what extent this structure resembles the one found in healthy subjects. Furthermore, we extract transient dynamical features via specific deviations from the stationary interrelation pattern. We find that (i) the UWS group is more heterogeneous than the two groups of healthy subjects, (ii) also the EEGs of the UWS group contain a stationary cross-correlation pattern, although it is less pronounced and shows less similarity to that found for healthy subjects and (iii) deviations from the stationary pattern are notably larger for the UWS than for the two groups of healthy subjects. The results suggest that the nervous system of subjects with UWS receive external stimuli but show an overreaching reaction to them, which may disturb opportune information processing.

## Introduction

The dynamics of the human brain is supposed to be profoundly non-stationary [1], as it is constantly exposed to ever changing environmental conditions, it receives and reacts continuously to external stimuli, and it monitors and controls equally changing internal processes. Each of these actions requires specific, well-orchestrated spatiotemporal synchronised activity of neuronal populations. Groups of neurons synchronise their production of action potentials, can trigger avalanches that spread across specific regions of the cortex, these avalanches can split into smaller groups initiating further avalanches or subside altogether, creating space for new spreads to occur. Such scenarios arise continuously, so that the same groups of neurons can be involved in the performance of different tasks [2]. For example, neurons of the sensorimotor system have been shown to be involved in selective attention as well as anticipatory mechanisms [3, 4]. This multimodality of certain neuronal groups promotes the variability of brain dynamics and increases the possibility to generate different patterns of synchronised neuronal populations. In fact, brain dynamics permanently generate the largest possible number of different spatio-temporal structures of synchronised neuronal activity, expressed by power laws [5–12]. All events, both the smallest and the largest possible, occur. This is true for both, the distributions of spatial sizes as well as that of the lifetimes of these patterns. Note, power law probability distributions (*P*(*x*)~*x*^-β^) with *β* ≥ −1 do not have an average value. Thus, the distribution of the spatio-temporal structures of ongoing synchronised neuronal activity do not have a typical scale, viz. it is scale free.

Is this enormous dynamic richness the product of an increased non-stationarity imprinted in brain signals? At least the morphology of electroencephalographic (EEG) recordings may change drastically over time, when comparing e.g., different sleep stages [13, 14], open or closed eye conditions [15] or during pathological situations like e.g., an epileptic seizure [16].

Mathematically, the term "non-stationarity" refers to temporal changes in the topology of the associated phase space of the system under consideration [17]. Each point in this space uniquely characterises a dynamical state of a system that may be occupied at a given moment, i.e., each point is defined by the minimal amount of information required to fully describe the associated dynamical state [18]. Hence, the evolution of a system is described by a trajectory in its phase space, or equivalently, by the transition probabilities from one dynamical state to another, represented by locations in phase space. In general, a system does not visit all theoretically possible dynamical states, but its movement is restricted to a subset in phase space, usually termed by the attractor of the system [18]. If transition probabilities do not change, the structure in phase space remains constant. This is precisely the definition of stationarity [17]. Thus, enlarged dynamical variability could be due to time-dependent transition probabilities in phase space, i.e. the underlying phase space structure could change, implying temporal modulations of the attractor topology.

Alternatively, the observed large variability could also indicate dynamic proximity to a quasi-critical state (see [19] and references therein), which could also explain the disproportionately high energy consumption of the human brain even at rest. [20–23]. Task related activity leads only to an insignificant additional energy expenditure [24]. Hence, the high demanding ongoing activity is a stationary feature, and at least in terms of the energy balance, variations are tiny.

This raises the question if it is possible to identify further highly stationary features in multivariate recordings of brain activity that are assumed to be highly non-stationary? The answer is affirmative, given that several groups independently reported amazing stability of spatial interrelation pattern. In early studies [25, 26] cross-correlations between selected EEG contacts in women have been reported, that are not only stable over a period of up to 9 months but show additionally only small variations across subjects. Stable correlation pattern of slow cortical potentials has also been found in electrocorticography of epilepsy patients during wakefulness as well as slow wave and Rapid eye movements (REM)sleep [27]. In addition, the identified correlation pattern shows certain similarity to that of spontaneous fluctuations of BOLD signals, which already suggest a direct link between electrical brain activity and oxygen consumption.

Intracranial recordings of epilepsy patients were considered in a graph-theoretical analysis [28] and a variant of time-delayed mutual information to construct a directed functional network [29]. The authors report the existence of network templates that are stable over minutes, hours, and days.

Average functional cross-correlation networks before, during and after an epileptic seizure have been found to be topologically equivalent and also inter-subject similarity was surprisingly high, by means of extracranial EEG [30]. These results were confirmed when the functional networks of EEGs containing epileptic seizures, sleep EEGs from healthy subjects, and EEGs from young and older adults with open and closed eyes were compared [31]. The pronounced average spatial correlation structure, denoted as “stationary cross-correlation pattern”, was almost independent of the physiological state as well as the subject under consideration.

Stability of the functional network, constructed by linear as well as nonlinear genuine cross-correlations, was studied in [32]. While linear cross-correlations turned out to be surprisingly stable, the correlations estimated by non-linear estimators fluctuate considerably, and temporal stability but subject specific differences have been reported for fMRI-recordings [33].

Finally, in a combined EEG-fMRI study [34] the authors provide evidence of a strong link between the stationary cross-correlation pattern found in extracranial EEGs and the large-scale resting state networks identified in functional magnetic resonance imaging. Using the fluctuations around the stationary pattern as a predictor for fMRI-networks, the authors yield specificity values above 98%. They state that variations of the cross-correlation pattern in EEG and BOLD-fluctuations are different manifestations of the same phenomenon even when the temporal scales of the electrical brain activity and BOLD signals are strikingly different.

Hence, the zero mode of the brain dynamics consists of the permanent maintenance of a stable spatiotemporal pattern. This ongoing activity should be regulated by a control mechanism, possibly self-organised, causing pronounced spatial correlations. In [31, 34] it was argued that this strongly correlated ongoing activity not only sustains vitally important processes, but also ensures efficient information processing. This stable framework of extended spatial relationships simultaneously enables rapid coordination of local functional networks as well as large-scale integration and has been interpreted as a reflection of the dynamics on or near to the attractor in phase space. Consequently, transient responses to external stimuli, task-specific actions, or physiological states such as a particular sleep stage, may be encoded in possibly tiny but specific deviations from this stationary correlation pattern [31].

Thus, in this picture, the permanent generation of a large set of neuronal activity patterns may not be due to changes in phase space topology, i.e., non-stationary behaviour, but is generated by the dynamic proximity to a critical point, which on the one hand provides for large variability and on the other hand requires large-scale correlations. That raises the question of how far this condition is preserved in the case of certain brain damage.

Patients with unresponsive wakefulness syndrome (UWS/vegetative state) lose the ability to be aware of stimuli. In general, network functional connectivity is reduced in patients with consciousness disorders, which is expected due to disruption of extensive cortico-cortical and thalamo-cortical networks. Patients in a minimally conscious state (MCS) showed changes in functional connectivity over time, particularly in the gamma frequency range [35] and theta-alfa range [36]. While patients with UWS show a disruption of fronto-parietal coherence in response to sensory stimulation, indicating a lack of information integration [37]. Findings that are in line with major theories of consciousness [38–40]. However, some studies show the opposite pattern, increased functional connectivity in long-range brain networks for slow (1–4 Hz) and even fast (13–35 Hz) rhythms in UWS versus MCS patients [36, 41].

The loss of consciousness is also experienced during sleep, where the level of consciousness fluctuates across sleep stages, accompanied by specific changes in the neurochemical environment [42], and interactions among brain regions [43].

Here we analyse EEG-recordings of subjects with UWS during rest and while being influenced by acoustic stimuli and compare the results with a control group under the same experimental conditions as well as with another group of healthy subjects during night sleep. The aim of this paper is threefold: (i) We probe whether the group with UWS also shows a pronounced stationary cross-correlation pattern and to what degree this pattern resembles the correlation structure found in healthy subjects. (ii) Furthermore, we investigate to what extent the deviations from the stationary pattern of the UWS group are affected by external stimuli compared to the control group iii) Finally, we explored whether this state shows dynamic similarities to sleep stages of healthy individuals.

## Materials and methods

Here we consider a group of 13 severely brain-injured patients who met the criteria of persistent vegetative state (UWS) [44] in comparison to a control group of 13 clinically healthy subjects without a statistical difference in age. Subject information is presented in Table 1.

**Table 1:** Information of UWS patients and subjects of the corresponding control group. We do not encounter a significant difference in age according to a Kruskal-Wallis rank sum test. m = male; f = female.

Subject	UWS Age/Sex	Control Age/Sex
1	45 f	38 m
2	46 m	45 m
3	46 m	47 m
4	55 m	54 f
5	57 m	56 m
6	61 f	59 f
7	61 m	60 f
8	62 f	60 f
9	62 f	60 f
10	66 m	62 m
11	67 m	66 m
12	72 f	72 m
13	80 f	72 m
Mean (+ /- s.d.)	60 (10.37) 7 m	57.77(9.98) 8 m

Additionally, we consider 10 young healthy male subjects between 21 to 31 years during night sleep (for subject details consult [31]). Electroencephalographic data (EEG) have been recorded in two different laboratories. Z.MT. had access to information that could identify individual participants during and after data collection.

### Data acquisition from UWS and control group

Continuous EEG was recorded from 19 scalp sites according to the International 10–20 system using an electrode cap with tin electrodes, referenced to A1-A2 with a Micromed polygraph (model SAM 32 FC1 LED) 16-bit amplifier at Santa Viola Hospital in Bologna, Italy. Electrooculogram (EOG) for vertical and horizontal eye movements was recorded from electrodes above and below the right eye, and the outer canthi of each eye in diagonal. All impedances were kept below 10 kΩ. The signals were recorded with digital band pass of 0.01–500 Hz at a sample rate of 1024 Hz and stored for off-line analysis. Finally, EEG was carefully inspected, and only artefact-free segments were accepted for further analysis.

The protocol was approved by the local Ethical Committee and medical staff of the Ospedale Santa Viola, Italy and followed the ethical standards of the Declaration of Helsinki [45]. The use of the data was authorised by means of written informed consent of the healthy subjects (controls) or legal caregivers of the UWS patients.

### Acoustic stimuli

A different temporal stimulus structure was presented during two conditions: rhythmic and arrhythmic. The auditory stimulation consisted of pseudorandom presentation of 400 stimuli, 80 of them corresponding to the subject’s own name, 80 were the control name matching in syllables number of subject´s name, and 240 corresponding to a standard name. All stimuli were recorded by the same male voice with a mean duration of 600 ms (+/-150) and replayed binaurally through headphones. In the rhythmic one the inter-stimulus interval was fixed at 1500 ms, during the arrhythmic condition four different intervals between stimuli were employed: 800, 1000, 2000 and 2500 ms. No response was requested.

### Sleep data

Sleep EEGs were recorded from 10 right-handed clinically healthy subjects during night sleep in the Sleep Laboratory of the Faculty of Psychology of the National Autonomous University of Mexico between 2007 and 2009. All subjects slept two nights at the laboratory, the first for adaptation to recording procedures and the second for EEG analysis. The protocol was approved by the Ethical Committee of the Faculty of Medicine of the National Autonomous University of Mexico and followed the ethical standards of the Declaration of Helsinki [38]. Standard polysomnography (PSG) and a standard scalp EEG were recorded using A1 as a reference electrode. For data acquisition, digital filters were set at 0.1 and 70 Hz for EEG, at 10 and 70 Hz for EMG, and 0.3 and 70 Hz for EOG. All night PSG data were digitised and stored with 1024 Hz sampling rate. Wakefulness and sleep stages were identified by an experienced researcher (M. C-C.) according to standard procedures using 30-sec epochs [46]. Detailed description of the sleep data can be found in [31].

### EEG analysis

For all EEG signals corresponding to UWS, Controls, and Sleep, electrodes Fp1, Fp2, F7, F8, T5, T6, O1, and O2 were excluded due to electrical noise or movements in some subjects. The EEG records were free of artefacts after careful visual inspection.

All EEG signals were downsampled to 128 Hz. The electrodes used for the study were F3, F4, C3, C4, P3, P4, T3, T4, Fz, Cz, and Pz to maintain the same set of electrodes in all healthy subjects and patients, and the signal was filtered to obtain a broad band between 1 and 30 Hz with a fourth-order Butterworth filter, with a low-pass of 30 Hz and a high-pass with a 1 Hz cut-of digital filters. Activity in the gamma frequency range was not analysed to avoid strong influence of muscle activity, as control subjects were awake, and some patients presented spasticity. Successively, the signals were re-referenced to the median [47].

For each subject of either group three-minute segments are considered. These segments consist of the first three minutes of either condition for the UWS and its respective control group. For the sleep recordings three-minute segments are constituted via randomly chosen 10 second windows, separately for each subject and each sleep stage. We accessed data for the present study in 2019.

**Fig 1:**
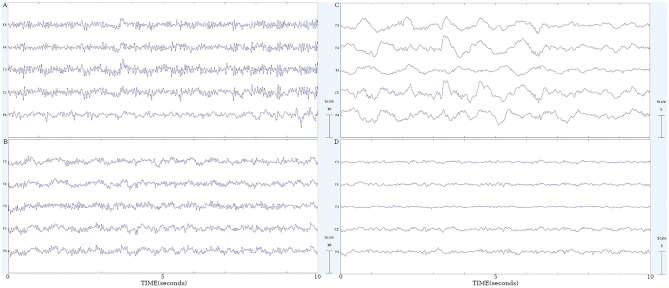
10-second EEG segments of 5 electrodes. **A)** one representative UWS patient; **B)** one subject of the control group during rest; **C)** one healthy subject during deep sleep and **D)** during REM sleep (see [31] for the description of the sleep subjects). The electrodes were selected to provide a representative image of the scalp.

To provide a visual impression of the quality of the collected EEG-data and to document the strong morphological changes during night sleep we provide in Fig 1 a collection of 10 second segments of 5 electrodes.

### Quantitative analysis

For the k^th^ 2 seconds segment of T data points, the zero-lag cross-correlation matrix is constructed:

$$C_{ij}^{\left(k\right)}=\frac{1}{T}\sum_{t=1}^{T}{\tilde{X}}_{i}^{k}\left(t\right){\tilde{X}}_{j}^{k}\left(t\right)$$ (1)

where ${\tilde{X}}_{i}^{k}\left(t\right)$ and ${\tilde{X}}_{j}^{k}\left(t\right)$ denote the signals of the k^th^ segment, measured by electrodes *i* and *j* respectively and normalised to zero mean and unit variance, viz.

$${\tilde{X}}_{i}^{k}\left(t\right)=\frac{X_{i}^{k}\left(t\right)-\;\langle X_{i}^{k}\rangle }{\sigma_{i}^{k}}$$ (2)

where $\left\langle X_{i}^{k}\right\rangle$ denotes the average and $\sigma_{i}^{k}$ the standard deviation of the raw signal evaluated for the k^th^ segment. We also estimated the nonparametric version of linear cross-correlations, namely Spearman correlations. As will be shown below, we obtain quantitatively similar results. Given that the usage of Pearson correlations is commonly used in the field of EEG analysis we focus thereafter on this quantity.

Here we are interested in correlation structures stable in time. Therefore, we estimate mean correlation matrices averaged over the three minutes periods for a certain condition (like e.g., rhythmic, arrhythmic, or e.g., a certain sleep stage) indexed by *l*:

$$\langle Cl\;ij\;\rangle =\sum_{k=1}^{K}C_{ij}^{\left(k,l\right)}$$ (3)

Alternatively, we also estimate for each subject the average correlation matrix over all conditions, like e.g., the average over all three-minute segments of all sleep stages:

$$\langle C_{ij}\rangle =\sum_{l=0}^{L}\langle C_{ij}^{l}\rangle$$ (4)

We denote this matrix as Stationary Pattern (SP) in the sequel.

Beside average cross-correlation structures we are interested in task specific deviations from a potential stationary structure. This is motivated by previous contributions [31, 34] indicating that transient features of the dynamics, like the response of a particular stimulus, is encoded in fluctuations or specific deviations from the stationary pattern. Therefore, we also estimate for each physiological condition the average deviation matrix as follows:

$$D_{ij}^{l}=\left|\left\langle C_{ij}\rangle -\langle C_{ij}^{l}\right\rangle \right|$$ (5)

To quantify similarity between two matrices we estimate Pearson correlations. To this end we sort the diagonal elements of each matrix in a vector, which is subsequently normalised to zero mean and unit variance. The scalar product of such two vectors, denoted by *ρ* in the sequel, serves as a measure for topological similarity. Significant differences of the correlation strength of two matrices we quantify via the non-parametric Mann-Whitney-Wilcoxon-rank test as well as the Kolmogorov-Smirnov test including Bonferroni correction for multiple testing.

**Fig 2:**
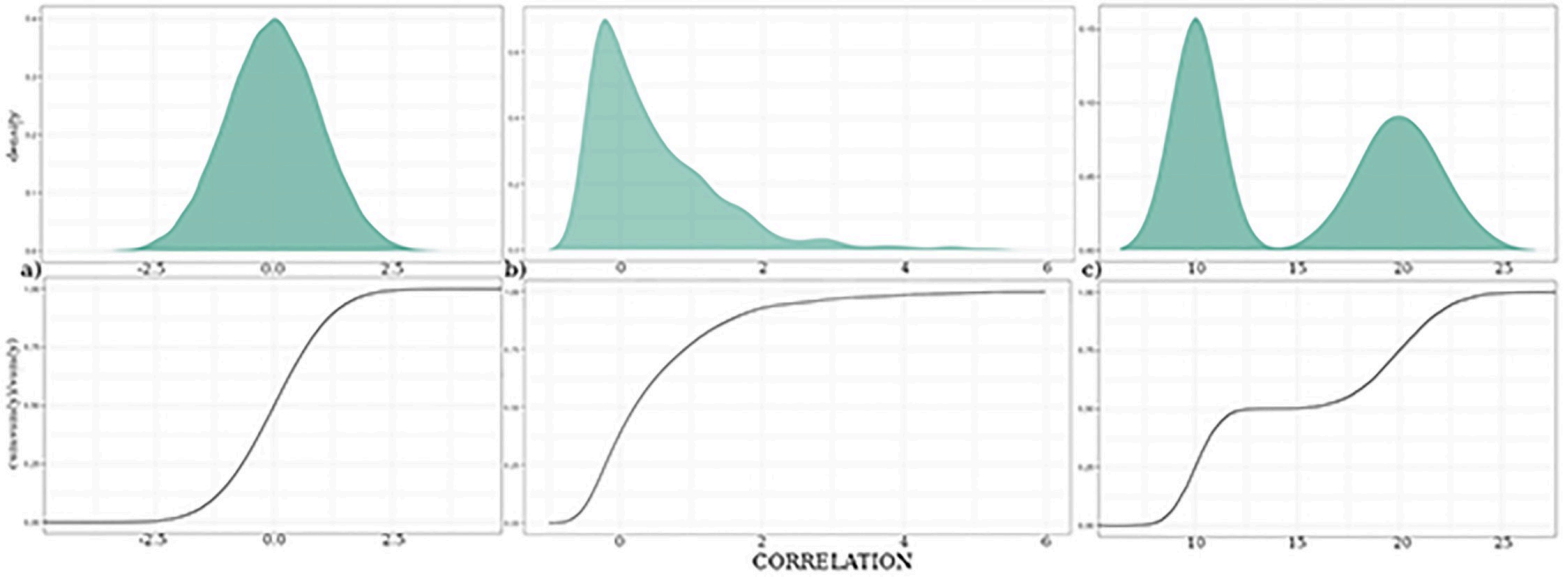
Different representations of probability densities as a histogram (upper row) and the corresponding cumulative probability distribution. **(a)** shows a symmetric, **(b)** an asymmetric, and **(c)** a bimodal distribution.

In the sequel we frequently display probability densities ρ(x) as a cumulative distribution $N(x)=\int_{-\infty }^{\chi }\rho (x\prime )dx$’ instead of using the traditional representation using histograms. The reason for this is that with small samples, as is the case in the present work, the visual impression of the histogram may be considerably distorted, whereas the cumulative distribution does not require any externally imposed discretization of the abscissa. However, given that the presentation of cumulative distribution is less common, we provide in Fig 2 a cartoon of a symmetric, an asymmetric and a bimodal distribution function, to facilitate interpretation of our numerical results.

We also estimated significance values for the comparison of different samples. To this end we employ the nonparametric Mann-Whitney-Wilcoxon rank test as well as the Kolmogorov-Smirnov test.

## Results

**Fig 3:**
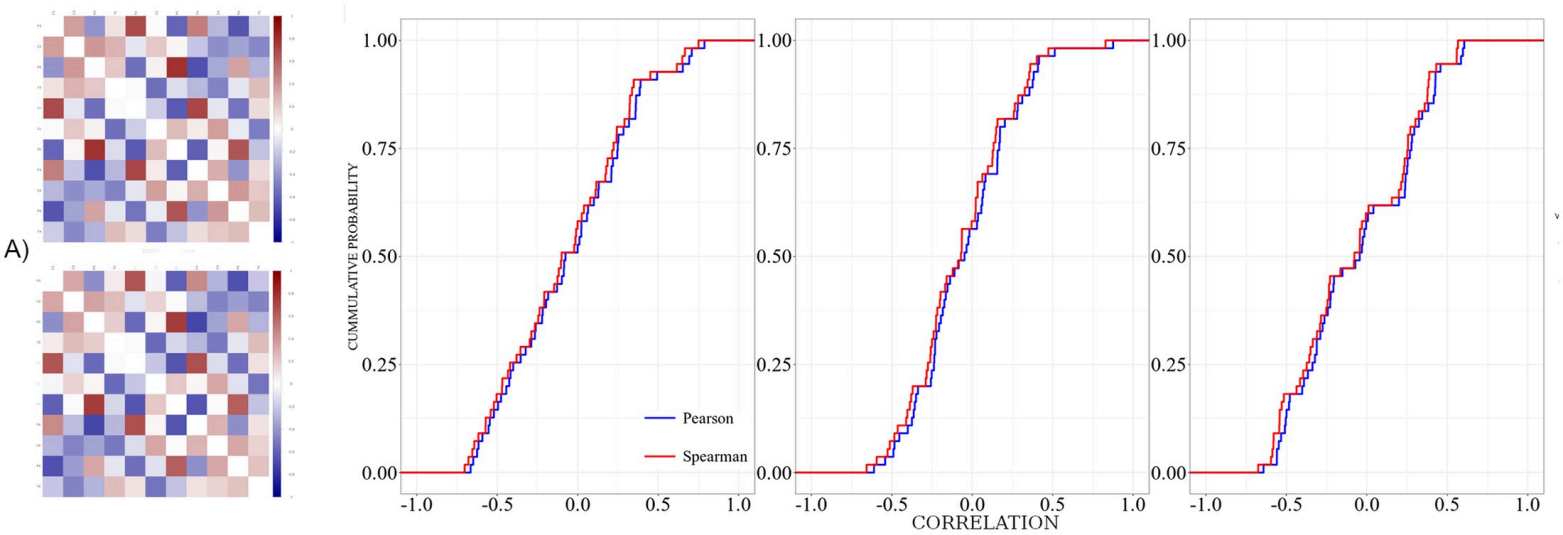
**(a)** shows interrelation matrices of one subject of the control group during rest using zero-lag cross-correlations (upper matrix) and Spearman correlation (lower matrix). **(b)**, **(c)** and **(d)** show cumulative probability distributions comparing both indices for one representative subject of the Control group (panel b), UWS group (panel c) during rest, as well as one subject during sleep (panel d). In all cases we show the stationary pattern, viz. the interrelation matrices averaged over the whole recording.

In a first step, we probe whether we obtain notable differences using Pearson or Spearman correlation for the construction of the functional network. To this end, we generated interrelation matrices using estimators for both indices and evaluate quantitatively the similarity of both schemes. Fig 3 displays corresponding results:

Upon inspection by eye, we find a striking similarity between the matrices derived for the Pearson and the Spearman correlation. This impression is confirmed quantitatively by applying the nonparametric Mann-Whitney-Wilcoxon rank test, as well as the Kolmorov-Smirnov test. In both cases we do not observe significant differences, p-values are in both cases notably above 0.3. Thus, in the sequel we employ exclusively Pearson correlations.

**Fig 4:**
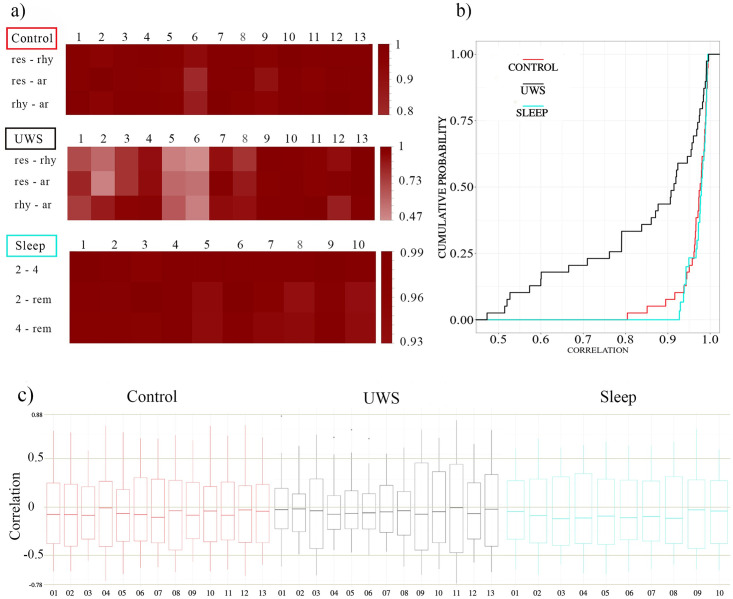
**(a)** Similarity *ρ* between correlation matrices averaged separately for each condition (res = resting, rhy = rhythmic, ar = arrhythmic as well as sleep stages 2, 4 and REM) and each subject: top row controls, centre UWS and bottom sleep. **(b)** Cumulated probability distributions of the similarity values *ρ* shown in panel (a). The red curve corresponds to the similarity values of the 13 participants of the control group, cyan to those of the 10 sleep EEGs and black to the 13 UWS. **(c)** Boxplots of the non-diagonal elements of the stationary pattern separately for each subject. Colours as in panel (b).

Next, we probe whether we encounter similar correlation structures for the same subject in different physiological conditions. Fig 4a and 4b display the results for each of the three groups. We note that the similarity for the two groups of healthy subjects is extraordinarily high. For the control group all Pearson coefficients *ρ* are above 0.8, while the lower limit for the similarity of the average correlation matrices of different sleep stages is even higher with *ρ*> 0.93. Here we provide a pairwise comparison of sleep stages 2, 4 and REM. In particular deep sleep (stage 4) and REM sleep show profound morphological differences with strikingly different power spectra. Nevertheless, the topological similarity of the spatial correlation structure is surprisingly high. Note the similarity of the cumulative distributions of the control group and the sleep EEG displayed in Fig 4b. Applying a Mann-Whitney-Wilcoxon rank test results in a p-value of p = 0.67, i.e., no significant difference.

For the UWS subjects, however, the lower limit is drastically reduced to 0.47, although also for this group about 50% of the comparisons lead to similarity values above 0.9, an extraordinarily high value. The fact that the Pearson coefficients for the comparison of the UWS group are distributed over a wider range indicates greater heterogeneity within this group. A quantitative comparison of the cumulative distribution of panel b confirms that the curve for the UWS-group is significantly different from the others (p<10^−4^) according to the Mann-Whitney-Wilcoxon-rank test (p<10^−5^, according to a Kolmogorov-Smirnov test).

This is confirmed by the results drawn in Fig 4c, where the distribution of the non-diagonal elements of the corresponding stationary patterns is represented in a box-plot format.

In all cases, median values are slightly shifted toward negative values. For both the control group and the sleep EEGs, we observe a comparatively broad distribution of the averaged cross-correlation values, i.e., a considerable number of cross-correlation coefficients deviate strongly from zero. This seems surprising at first glance, considering that drastic changes in EEG signals can be observed at least throughout the night’s rest. Also, the records of the UWS and its control group are supposed to be highly nonstationary, given the changing conditions during the conduct of the experiment. Therefore, one also might expect the values of the cross-correlation to vary greatly over time and to change sign, so that the average values over longer time intervals should lead to estimates close to zero. Instead, the magnitude of cross-correlation of the sleep EEG varies within ±*0*.*5* and those of the control group even within ±*0*.*75* Furthermore, we observe that the widths of the distributions of the mean correlation coefficients remain almost constant, within each of the two groups of healthy subjects.

**Fig 5:**
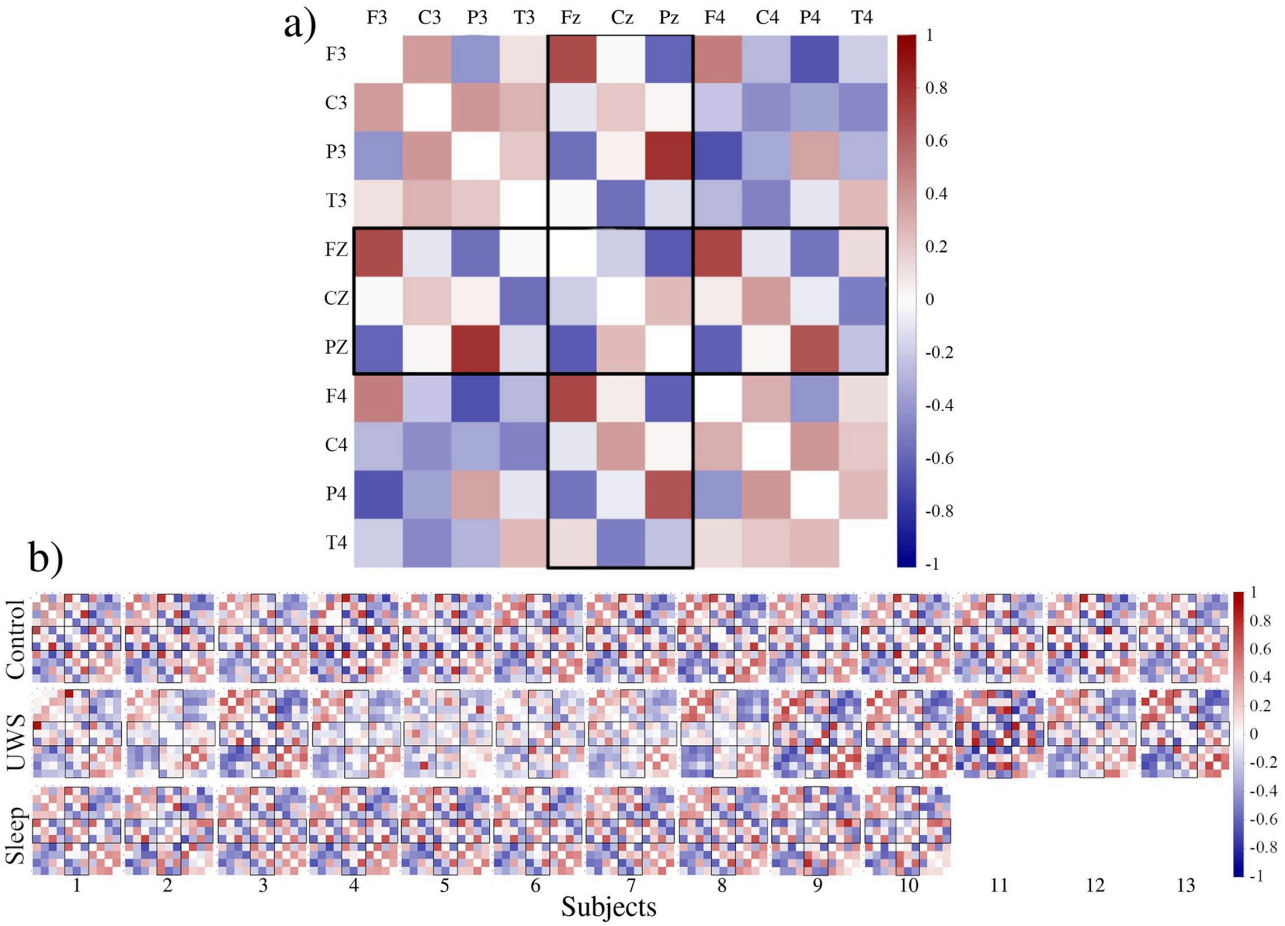
**(a)** Stationary correlation pattern of subject 1 of the control group. Electrodes are ordered in such a way that the upper left and lower right square of each matrix display intra-hemispheric cross-correlations of the left and right hemisphere respectively. The lower left and upper right square show inter-hemispheric correlations. The three-by-three central square contains the correlation coefficients between the central electrodes and the rectangles display the correlations between hemispheric and central contacts. The diagonal elements of each matrix are set to zero to improve visual impression. **(b)** Stationary correlation pattern of each subject considered in the present study. First row, control subjects, middle row UWS, bottom row sleep.

In contrast, the UWS group is quite heterogeneous. Occasionally the distributions are very narrow, as in the case of subjects 2, 4, 5 or 6, or the non-diagonal entries vary in the same wide range as in the control group (see boxplots of subjects 3, 8, 9, 10). Fig 5 provides a visual impression of the stationary pattern of all participants.

Just by eye revision of the stationary pattern displayed in Fig 5 we can state that (i) intra-hemispheric correlations are by trend positive, (ii) inter-hemispheric correlations are by trend negative and (iii) the spatial correlation patterns of the two groups of healthy subjects are conspicuously similar. Furthermore, we observe that within the UWS group the stationary pattern of some subjects is only weakly expressed (e.g., subjects 2 or 5), while some others like e.g., the pattern of subject 11, contains comparably large positive as well as negative entries. This qualitative observation confirms the results of Fig 4c.

**Fig 6:**
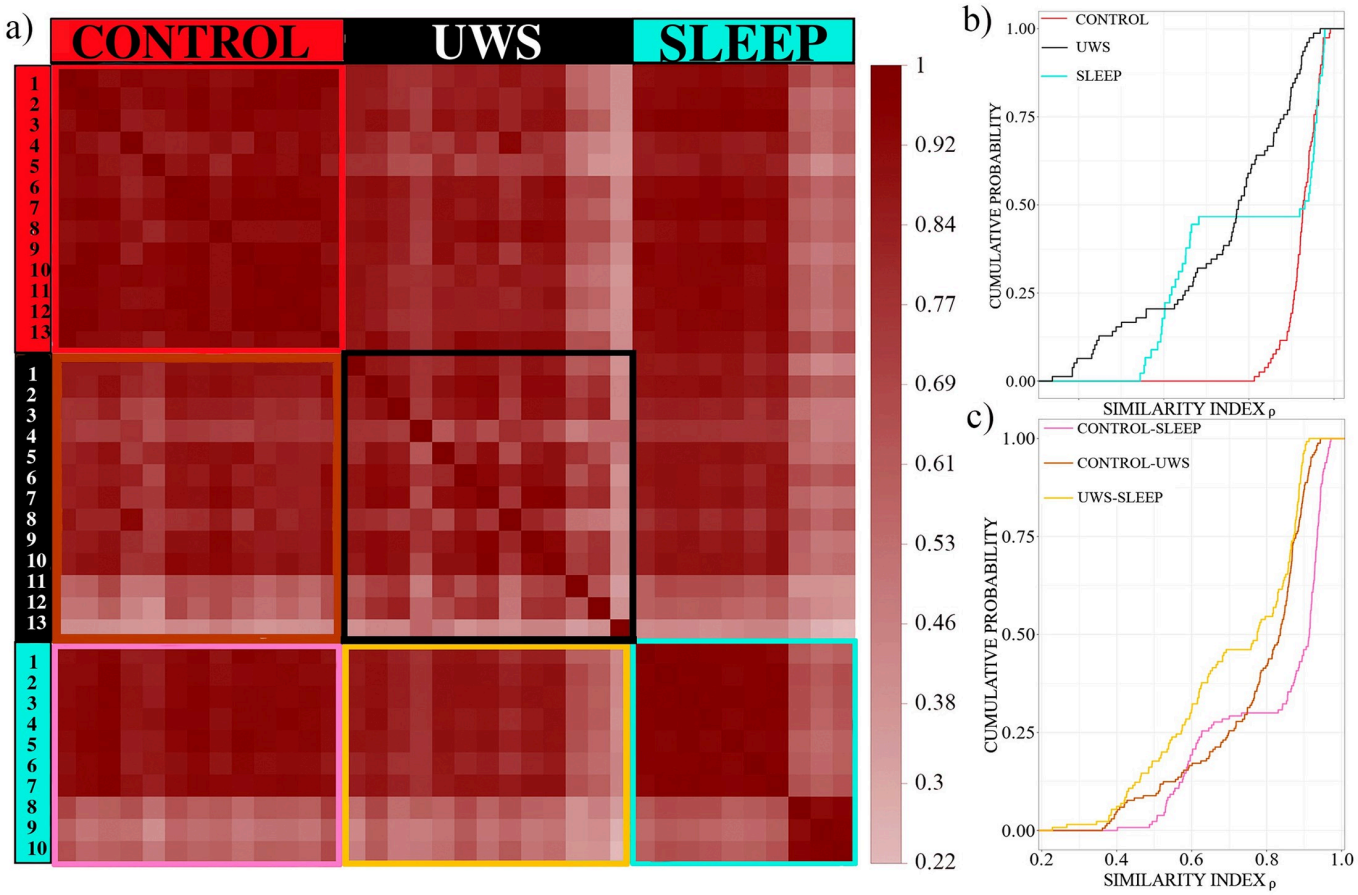
Comparison of stationary pattern within and between groups. **(a)** Colour coded similarity Index *ρ* or intra- and inter-group comparisons, **(b)** cumulative distribution functions for intra-group comparison, colour coding is the same as in Fig 4, **(c)** same as (b) for inter-group comparisons, pink indicates the comparison of the control and sleep group, brown UWS and control and yellow sleep with the UWS-group.

But not only the correlation strength of the pattern, also the spatial distribution of the matrix elements of the UWS group is more heterogeneous. While several matrices are to a certain degree topologically like those observed for healthy subjects (for instance subject 3), other correlation structures deviate to a certain degree (see e.g., the pattern of subject 8 or 11). Fig 6 provides a more quantitative picture from this situation.

According to the results shown in Fig 6, the intra-group similarity of the control group (upper left square of the red frame) is strikingly high. All *ρ* values are above 0.8 (Fig 6b). Instead, the stationary pattern of the sleep EEGs (lower right square with the cyan frame) decays in two subgroups of subjects 1 to 7 and 8 to 10, which provokes the well-expressed horizontal plateau of the corresponding cumulative probability distribution in panel b. Within subgroup similarity is again extraordinary high with *ρ* values above 0.9 (right hand flank of the corresponding distribution function of Fig 6b), comparison between members of the two subgroups *ρ* takes values between 0.4 and 0.65 (left hand wing of the same distribution function).

The fact that the stationary pattern of the sleep EEG decays in two subgroups also provokes the pronounced shoulders of the cumulative probability distributions for the two comparisons with sleep EEGs, viz. distribution functions are bimodal (yellow and pink curves in Fig 6c). While the stationary patterns of the larger subgroup constituted by subjects 1 to 7 is highly similar to the control group (*ρ*>0.8), the similarity indices for the comparison with the smaller subgroup vary between 0.5 and 0.7. Nevertheless, these results still suggest strongly that the mean spatial correlation structure is almost the same for both groups of healthy subjects.

The stationary patterns of the UWS group, however, are much more heterogeneous. Similarity estimates are almost uniformly distributed between 0.2 and 0.95 for intra-group comparisons (the corresponding cumulative distribution of panel b follows approximately an inclined straight line) and also the similarity values for the inter-group comparisons vary over a large range (Fig 6c). We will have a closer look on group differences in the sequel.

**Fig 7:**
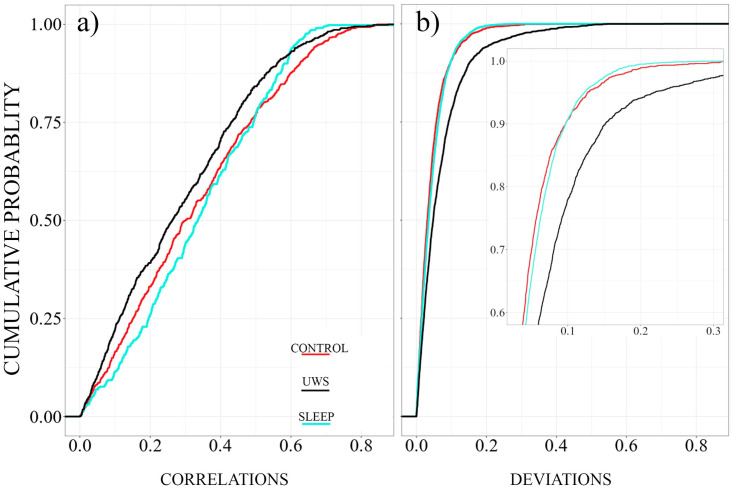
**(a)** Cumulative distribution of the absolute values of non-diagonal elements of the stationary patterns. **(b)** Cumulative probability distribution of the non-diagonal elements of the deviation matrices. The UWS patients are shown in black, the cyan colour corresponds to the sleep EEGs, and the results of the control group are drawn in red.

Fig 7a displays the cumulative distributions of the absolute values of the non-diagonal elements of the stationary patterns of the three groups. The absolute values of the average correlation coefficients are almost uniformly distributed between zero and approximately 0.85 (i.e. the cumulative distribution functions resemble almost a straight line). However, we notice some differences between the three groups. The distribution for the UWS is somewhat displaced toward lower values, centred at about 0.25, while the others take central values above 0.3, i.e., the patterns of the two groups of healthy subjects are more pronounced. However, while the UWS and the control group have a pronounced tail toward larger values, the distribution of the sleep EEGs is almost symmetric (see estimates of the first three moments listed in Table 2).

**Table 2:** First three moments of the cumulative probability distributions shown in Fig 7a.

	Average	Standard Dev.	Skewness
**Control**	0.33	0.21	0.34
**UWS**	0.29	0.2	0.47
**Sleep**	0.34	0.18	0.03

In all three cases we are confronted with non-stationary signals, due to different external stimuli or strikingly different sleep stages. In [31, 34] it was argued that the transient, viz. a nonstationary part of the brain dynamics is expressed by task or stimulus specific deviations from the stationary pattern. Thus, we are also interested to study properties of the deviation matrix *D* (Eq 5). The cumulative distributions of the non-diagonal elements are drawn in Fig 7b.

Now the distribution function of the UWS group shows a notably higher probability for the occurrence of larger deviations, while the curves of the two other groups are almost identical. The stationary pattern of the UWS group is on average less pronounced than that of the other groups but show significantly greater deviations from this pattern for different physiological states than the healthy participants.

**Fig 8:**
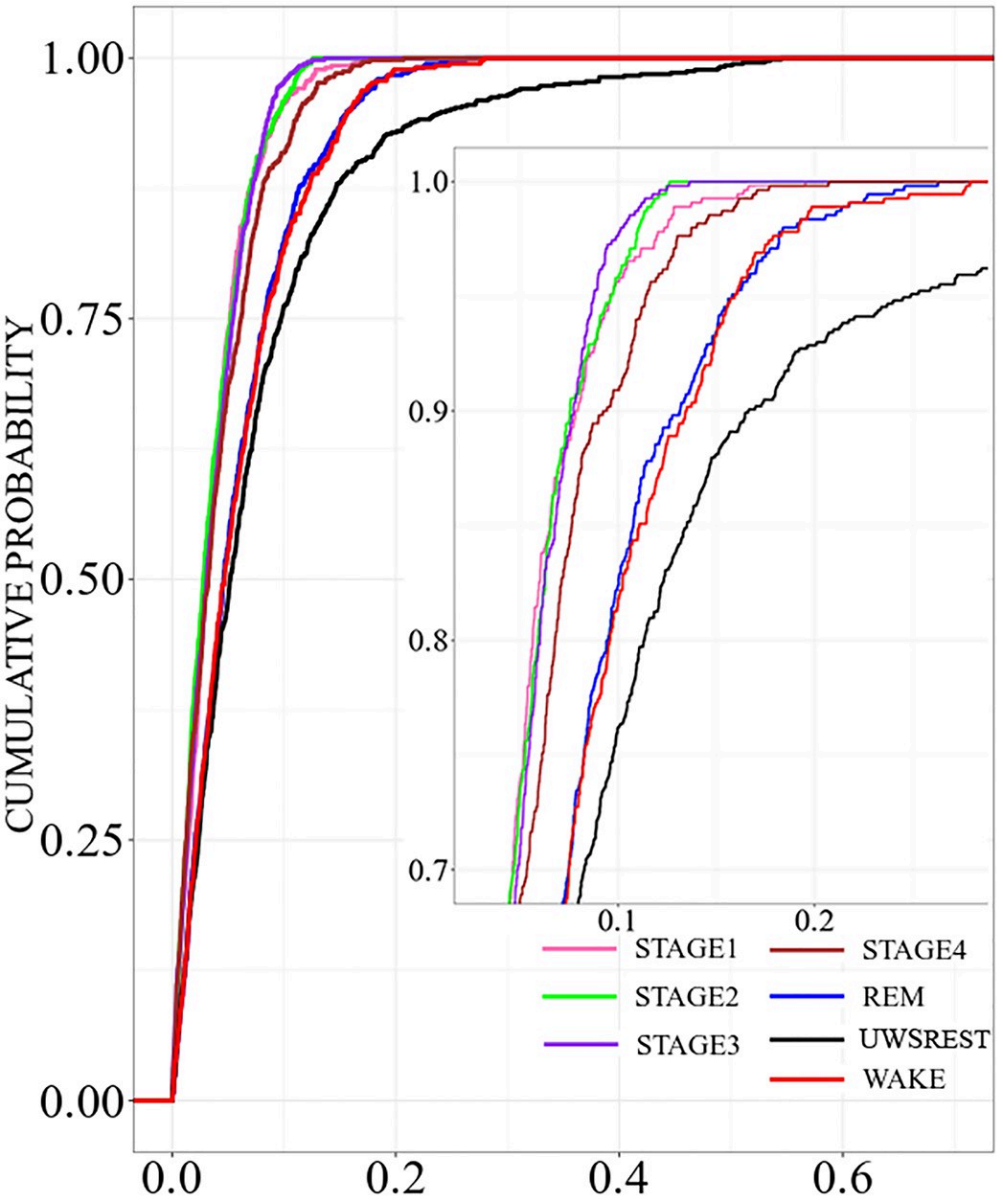
Cumulative probability distribution of the non-diagonal elements of the deviation matrices for the 10 sleep EEGs, separately for the different sleep stages as well as for the UWS in the resting state condition. UWS results are shown in black. Colour codes of the sleep stages are as follows: Awake condition (red), Stage 1 (pink), Stage 2 (green), Stage 3 (purple), Stage 4 (brown) and REM sleep (blue).

Next, we turn to the comparison with the sleep data to probe if the brain dynamics of UWS-patients has something in common with certain sleep stages of healthy people in terms of the deviations from the stationary pattern. Cumulative distribution functions of the non-diagonal elements of the deviation matrix for different sleep stages are drawn in Fig 8.

Smallest average deviations are observed for the transitional stages 1 and 3, as well as for light sleep stage 2. Their estimates are mainly below 0.1. Slightly larger deviations are observed for deep sleep (stage 4). For REM sleep and the awake state we encounter even larger estimates. The upper limit of their distribution functions is about 0.275. Both curves are almost identical, which fits to the observation that also their spectral content as well as the morphology of the EEG-signals is quite similar. REM sleep is considered an active state, and, thus, it is not unreasonable to assume that both vigilance states also have similar dynamic characteristics.

However, the distribution function obtained for the UWS-group deviates considerably. In comparison with those derived for different sleep stages, the deviations are most pronounced in this group. It has a strong tail toward large values with an upper limit somewhat above 0.5. Thus, dynamical features decoded in the deviations from the stationary pattern are strikingly different from each of the sleep stages of healthy subjects.

**Fig 9:**
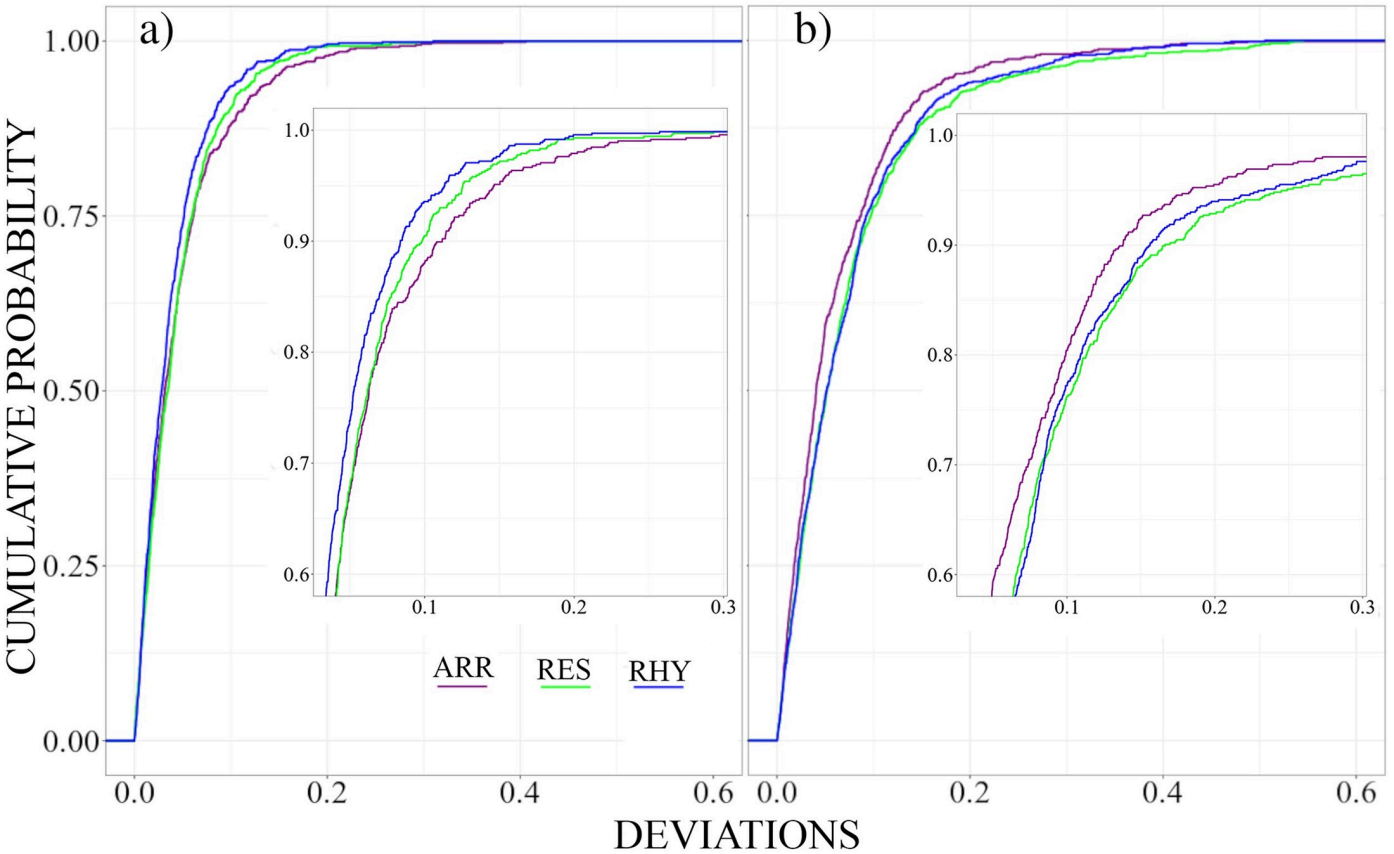
Cumulative probability distribution of the non-diagonal elements of the Deviation matrices for the Control **(a)** and the UWS group **(b)**. Deviations for the Resting condition are drawn in green, those for the Rhythmic and Arrhythmic conditions are drawn in magenta and blue respectively.

In what follows we compare more in detail deviations for the UWS and control group during rest and acoustic stimuli. Both groups were exposed to two different acoustic stimuli, a rhythmic and an arrhythmic one (beside the rest-condition without any stimuli). The rhythmic stimuli, which are somewhat monotonous, are easier to predict, process or, if necessary, suppress. Adequate processing of the arrhythmic stimuli is more demanding and penetrates the consciousness more easily. Thus, if specific deviations from the steady-state pattern reflect a transient dynamic [31, 34], one might assume intuitively that the more demanding arrhythmic stimuli elicit stronger deviations than the rhythmic ones. This is precisely the case for the control group (Fig 9). Arrhythmic stimulation provokes the strongest deviations from the steady state pattern, while the more predictable, rhythmically given impulses show even smaller deviations than the less controllable resting state.

For the UWS-group we encounter qualitatively different results than for the control group. Now the arrhythmic stimulation causes less pronounced deviations, as if the subjects of the UWS group do react less to the more complex stimulation. On the other hand, the rhythmic and resting state show somewhat similar characteristics, although the distribution obtained for the resting state shows a longer tail towards larger deviation values (enlarged positive skewness).

This visual impression is confirmed by the first three moments of the probability distributions shown in Table 3 and comparisons among conditions in Table 4. Average as well as skewness values for the UWS-group are systematically higher than those for the control subjects and also the widths expressed by the standard deviation of the distributions is larger.

**Table 3:** First three moments of the cumulative probability distributions shown in Fig 9.

Moments	Control			UWS		
	Average	Std. Dev.	Skewness	Average	Std. Dev.	Skewness
**Rest**	0.044	0.04	2.13	0.08	0.08	2.62
**Rhythmic**	0.040	0.04	2.25	0.07	0.07	2.29
**Arrhythmic**	0.050	0.05	2.53	0.06	0.07	3.15

**Table 4:** P-values according to the Kolmogorov-Smirnov (KS) test for the comparison of the three experimental conditions (rest, rhythmic, arhythmic) for the control and UWS groups.

P-values KS-test	Rest vs. Rhythmic	Rest vs. Arrhythmic	Rhythmic vs. Arrhythmic
**Control**	**0.0014**	0.687	0.0249
**UWS**	0.91	**0.0002**	**0.0006**

**Fig 10:**
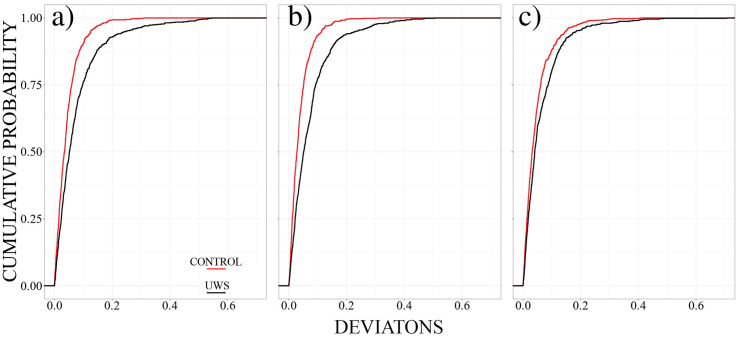
Cumulative probability of the deviation matrices for the Resting **(a)** Rhythmic **(b)**, and Arrhythmic **(c)** conditions. Control and UWS groups are displayed in red and black respectively.

Finally, we compare directly the UWS and control groups in either condition. Results are summarised in Fig 10, significance values derived from the Kolmogorov-Smirnov test as well as the first moments of the probability distributions are listed in Tables 5 and 6 respectively.

**Table 5:** P-values according to the Kolmogorov-Smirnov (KS) test for the comparison of the control *vs*. UWS groups.

P-values KS-test	Rest	Rhythmic	Arrhythmic
**Control vs UWS**	**6.55e-14**	**2.2e-16**	3.96e-02

**Table 6:** First three moments of the cumulative probability distributions shown in Fig 10.

		Average	Standard Dev.	Skewness
**REST**	**Control**	0.044	0.042	2.13
**UWS**		0.077	0.087	2.62
**RHYTHMIC**	**Control**	0.038	0.037	2.25
**UWS**		0.073	0.076	2.29
**ARRHYTHMIC**	**Control**	0.047	0.050	2.52
**UWS**		0.063	0.071	3.15

Fig 10 reveals that the deviations derived for the UWS-group are consistently larger in all conditions. Largest differences between both groups are obtained for resting state and rhythmic stimulation, but in all cases, the cumulative distribution shows a notably longer tail toward larger deviation values. Hence, not only the stationary pattern of spatial cross-correlations is affected by the unresponsive wakefulness syndrome, but also transient dynamics as manifested by specific deviations from a stable scaffold of linear correlations.

Notably, the difference between the arhythmic mode between the control and UWS group is not significant on a 1% significance level. In this condition the control group showed largest deviations while the UWS group reacted less in this setting. Hence, both probability distributions get closer.

Table 6 provides a quantitative impression for these comparisons. Average values as well as standard deviations are consistently larger for the UWS group in all conditions and also the asymmetry parameter shows a longer tail toward larger values for the UWS-group. Thus, deviations from the stationary correlation structure are consistently larger for the UWS-subjects. However, while average values are nearly double for the US in during rest and rhythmic stimulation, differences are reduced for the arrhythmic setting and also the standard deviation takes the lowest value for this condition. Hence, UWS patients show abnormally strong responsivity to any kind of external stimuli while, at the same time, they process these distinct stimuli in a less differentiated way. These are typical signs of a dynamically unstable system showing weak stationary and therefore a debilitated stable spatiotemporal structure and dynamics [48].

## Discussion

A very useful research approach in the field of neurology and cognitive neuroscience is to distinguish between intrinsic self-generated brain activity from activity associated with processing of external stimuli. We addressed this question by exploring the intrinsic connectivity brain dynamics in UWS patients, a control group, and normal sleep.

Hereby, the entire study relies on the Pearson coefficient as a bivariate measure to construct the functional network with the aim to extract and characterise a stable, pronounced correlation pattern covering the whole scalp. However, zero-lag correlations are susceptible to volume conduction [49, 50], which can also induce pronounced cross-correlations that could mimic strong, temporally stable interactions even between distant electrodes. This issue was extensively discussed in [30], where a similar analysis of 20 extracranial recordings of patients with temporal lobe epilepsy were presented.

To prove that the observed stationary pattern is not due to volume conduction, two different strategies have been followed. At first the authors of [30] repeated their numerical analysis using lagged cross-correlations. To this end they searched for maximal (positive or negative) cross-correlations, while varying the time lag by which one signal was shifted relative to the other. In a second attempt, they applied the so called “weighted phase lag index” [51], which is an improved version of the imaginary part of the coherency [50]. Since volume conduction activity affects only the real part of the cross spectra, measures based solely on the imaginary part of the Fourier components are immune to such contamination. In both cases the authors were able to reproduce their results almost quantitatively (see e.g. Fig 7 of [30]). In view of these results, we are confident that also in the present case we evaluate genuine interrelations of brain activity measured at different locations.

Our results show that UWS patients have an overall steady-state correlation pattern similar to that of healthy subjects, while acoustically stimulated during wakefulness and during sleep. The observed stationary pattern was less pronounced in UWS than healthy subjects, and more heterogeneous among UWS patients in strength and spatial distribution. The degree of similarity for the same subject in different conditions was above 0.8 for control subjects, 0.93 for healthy subjects during sleep and only 0.47 for UWS. While the drop in similarity for the UWS group is considerably large, still 50% of the values are above 0.9 (Fig 4).

A possible source of the more heterogeneous results obtained for the UWS patients might be the different clinical histories of the subjects, including differences in the location of brain damage. In general, UWS patients are characterised by a complex pattern of structural and functional brain damage. UWS is often the result of diffuse brain damage to white matter, with contributions from neuronal loss in the thalami and the hippocampus [52]. Impairment of top-down connections, particularly from frontal to temporal cortices, is also a key factor in this condition [53]. The thalamus, which is critical for cognition and awareness, is disproportionately damaged in these patients. Also, long-range cortico-cortical and cortico–thalamo–cortical functional disconnections are observed in UWS patients [54, 55], giving rise to numerous diverse patterns of activity, which is underpinned by damaged brain anatomy. Thus, these massive structural changes in the UWS group might contribute to the heterogeneity observed in the stationary pattern.

Another source of heterogeneity could be the influence of varying heart rate, breathing or any kind of muscle artefacts, although we took care to analyse exclusively segments that are free from artefacts, at least by eye revision. In particular, such artefacts should significantly contaminate both sleep EEGs and those signals containing epileptic seizures. Heart rate, as well as breathing changes notably for different sleep stages [55–61] and of course during the peri-ictal transition of epileptic crisis [62, 63]. Nevertheless, neither for the sleep EEGs [31] nor for recordings of epileptic patients we could detect major influences of such activity but observe an extremely high similarity of the corresponding stationary pattern [31]. Therefore, we believe that this issue is also less important in the case of the UWS-patients.

As previously reported [31], the stationary correlation pattern was present during whole night sleep despite its large variability in power, oscillatory activity, and level of consciousness characteristic across sleep stages. Again, the distributions of the absolute values of the non-diagonal elements of the stationary correlation pattern show that the values obtained for UWS are on average somewhat lower than those obtained for the control and sleep groups (Fig 7a). These results confirm that even patients presenting severe brain injuries with UWS show an organised, stable connectivity pattern that is independent of the state of consciousness.

Regarding the characteristic deviations from this steady-state correlation pattern, a different picture emerged. Here, we observed smaller deviations from the stationary correlation pattern for control and sleep groups than for UWS patients (Fig 7b), with non-REM sleep in healthy subjects showing significantly smaller deviations than REM sleep and wakefulness (Fig 8).

Comparing the deviations during the different settings of acoustic stimulation, the control group showed lower deviations during the rhythmic stimulation than in the resting and arrhythmic condition, whereas USW patients showed the opposite response, namely higher deviations in the resting and rhythmic settings than in the arrhythmic stimulation (Fig 9 and Table 5). Comparison between groups shows that deviations were consistently greater in UWS patients compared with control subjects under the three conditions-resting, rhythmic, and arrhythmic stimulation.

It is known that in humans [64], monkeys [65], and rats [66], the number of metabolic brain activity patterns is greater when awake than when consciousness is impaired by anesthesia. Furthermore, loss of consciousness following severe brain injury is associated with a reduction in dynamic patterns and functional connectivity. Under these conditions, the balance of integration and segregation of neuronal populations is disturbed and thus a proper functioning of brain activity, for example during the processing of external stimuli or cognitive processes, is no longer guaranteed [67, 68]. In addition, deactivations of the default mode network [69] and impairments in connectivity have been reported during sleep and after severe brain injury [70–72]. Notwithstanding, a pattern of metabolic brain activity remains stable in different states of consciousness under the effect of general anesthesia [64–66, 73–75]. The maintenance of this connectivity pattern has been attributed to the anatomical scaffold that remains unchangeable [63, 76, 77].

Nevertheless, our results show that even after severe brain injury that extinguishes or at least severely impairs consciousness, the electrical activity of neurons remains structured in a very similar functional connectivity pattern as in healthy subjects during wakefulness and sleep, suggesting that the anatomical structures involved in consciousness are not causally involved in the dynamical baseline brain state. This is also consistent with the observation of the same connectivity pattern in steady state under a pathological condition that drastically alters brain activity, namely an epileptic seizure [30, 31]. Such a robust topology of the connectivity pattern is characteristic of the dynamics on the attractor in phase space (see appendix of [31]).

All these findings suggest that this is a pattern of background activity that could underpin both, the autonomic nervous system functions present in UWS, sleep (and epilepsy), and the structure of the interrelated activity required for consciousness to emerge. As it is addressed in theoretical-experimental proposals in which not only the level of consciousness is contemplated, as in the present study, but the meaning of the experience that the interaction with the environment entails (see [40, 78, 79]).

Although the correlation structure is stable for each of the subjects in the UWS-group, the patients show greater heterogeneity both in the magnitude and the spatial distribution of the average cross–correlations. This heterogeneity may be explained by differences in the etiology of the UWS, brain damage, and drug treatments of the patients.

A way to extract the stimuli-induced activity or complex non-stationary structure of the EEG signal that accompanies the sleep stages is by analysing the deviation from the stationary correlation pattern that occurs during the different conditions. During sleep, healthy subjects showed higher deviations for the active states (REM sleep and wakefulness) than slow wave sleep and light sleep, possibly due the greater information flow that is needed in the active states. Unexpectedly, UWS patients showed even higher deviations than healthy subjects in active states. This result supports the idea that there is a fine-tuned balance between the integration and segregation of information to interact appropriately with stimuli. It has been demonstrated that the optimal level of GABAergic inhibition is needed for the generation of dynamical richness [80] and efficient cognitive activity [81].

Specifically, Crone et al. [82] found reduced inhibition and increased oscillations in in UWS patients compared to controls and minimally conscious state in brain areas of the default mode network (DMN), a group of regions that consistently show greater activity in resting state than during a task [83]. In a previous work of simultaneous EEG and fMRI during resting state, Arzate-Mena et al. [34] applied the EEG deviations as a predictor for the general linear model to the fMRI data showing nodes of activity corresponding to the DMN.

Taken together this evidence with the exaggerated deviations found in the UWS patients in the present study may indicate that the DMN is altered, leading the system to an unstable operational mode that affects its interaction with the surrounding stimuli. This observation is confirmed by the stimuli-induced connectivity pattern of deviations analysed during each acoustic context.

Healthy subjects present the strongest deviations in the arrhythmic condition and similar values between rest and rhythmic condition. When the brain is stimulated in a rhythmic time structure, it is easier to predict and adapt to suppress information that does not represent consequences and does not require responses as subjects passively listen to the stimuli. While arrhythmic stimulation is probably more intrusive and difficult to suppress, which demands a greater involvement of stimulus monitoring.

In contrast, UWS patients showed stronger deviations in the rhythmic and resting conditions than arrhythmic acoustic stimulation, but importantly, closer to normal levels. These results provide evidence that these patients might transiently interact with external elements if there is a pattern of stimulation that constantly excites the system and does not allow adaptation to its usual dynamics; however, this hypothesis requires further experimental verification.

In conclusion, patients with UWS showed a dynamic stimuli-dependent transient network fused into structure of interactions between areas with a reduced level of correlations and anticorrelations compared to healthy subjects, which prevent them from having an optimal activity to notice the stimuli and to be able to interact with them.
